# Racialized disparities in pain and pain care among Belgian youth

**DOI:** 10.3389/fpsyt.2025.1579144

**Published:** 2025-07-01

**Authors:** Ama Kissi, Sean Carey, Dries Debeer, Dimitri M. L. Van Ryckeghem, Adam Hirsh, Tine Vervoort

**Affiliations:** ^1^ Department of Experimental-Clinical and Health Psychology, Faculty of Psychology and Educational Sciences, Ghent University, Ghent, Belgium; ^2^ Department of Psychology, Indiana University Indianapolis, Indianapolis, IN, United States; ^3^ Faculty of Psychology and Educational Sciences, Ghent University, Ghent, Belgium; ^4^ Department of Clinical Psychological Science, Maastricht University, Maastricht, Netherlands; ^5^ Department of Behavioural and Cognitive Sciences, University of Luxembourg, Esch-sur-Alzette, Luxembourg

**Keywords:** perceived discrimination, racism, pain care, children, pain tolerance, pain intensity, pain frequency

## Abstract

**Introduction:**

Research highlights racialized inequities in pain and pain care, yet the experiences of youth–particularly in Europe–remain largely understudied. The current study addressed this gap by examining differences in perceived racialized discrimination in pain care and pain outcomes (i.e., pain intensity over the past two weeks and six months, pain frequency over the past six months, and pain tolerance) among Black/Brown and White youth in Belgium. Additionally, we explored whether perceived racialized discrimination mediated the relationship between racialized identity and pain outcomes.

**Methods:**

Seventy-six youth (52 girls, 17 boys, 2 non-binary individuals) aged 8-17 (*M*_age_ = 15.17; *SD_age_* = 2.48) completed a cold pressor task to assess pain tolerance. Participants also reported their experiences of racialized discrimination in pain care, their pain intensity over the past two weeks and six months, and their pain frequency over the past six months.

**Results:**

Results indicated that Black/Brown youth reported greater perceived racialized discrimination in pain care and demonstrated lower pain tolerance than White youth. No significant group differences were observed for the other three pain outcomes. Perceived racialized discrimination in pain care only mediated the relationship between racialized identity and pain intensity over the past two weeks.

**Discussion:**

These findings suggest that racialized disparities in pain and pain care exist among youth living in Belgium. However, given the relatively small sample size, the results should be interpreted with caution. Additional research on racialized disparities in pain and pain care among youth using larger and more diverse samples is warranted.

## 1 Introduction

Pain is a common experience (1–5), with accumulating evidence highlighting significant racialized differences in how pain and associated suffering is experienced. For instance, systematic literature reviews and analyses of studies using experimentally induced pain to assess racial and ethnic disparities in pain sensitivity suggest that Black individuals tend to exhibit lower pain tolerance and report higher pain intensity relative to their White counterparts (6, 7). Additionally, clinical studies reveal that, compared to White individuals, Black individuals experience more frequent and higher levels of clinical pain (e.g., chronic pain and arthritis), which are associated with greater functional disability and suffering (8–12). These findings challenge the faulty belief that Black individuals experience less pain than White individuals–a misconception that persists even among individuals with medical training (13–15).

Findings of worse pain experiences among Black individuals compared to White individuals are particularly worrisome, as research has also demonstrated that Black individuals are less likely to receive quality pain care compared to White individuals (9, 12, 16, 17). Indeed, according to a review by Anderson and colleagues (9), Black individuals are less likely to receive adequate diagnostic assessments and analgesics, and are more likely to have their pain underestimated. For example, a prospective study of individuals with chronic back pain (18) showed that, despite the fact that Black patients had higher pain levels and worse functional disability than White patients, providers tended to underestimate their pain and were less likely to attribute it to a serious underlying cause. Black patients were also less likely to receive radiography or advanced imaging studies to investigate potential underlying causes of their pain; this pattern remained even after controlling for relevant factors such as indicators of socio-economic status (i.e., income, insurance status, education) and pain severity. Furthermore, a study by Todd and colleagues (19) showed that disparities in pain care also extend to treatment decisions. Specifically, they found that Black patients presenting to the emergency department with long-bone fractures were 66% more likely than White patients to not receive analgesics.

Furthermore, evidence indicates that these inequities in pain care are widespread and concerning, as they appear across various age groups (i.e., adults and children), treatment settings, and pain conditions, and may directly and indirectly contribute to pain and other health problems (e.g., depression and cardiovascular disease) among Black individuals (9, 10, 20). More specifically, such adverse health outcomes may arise through suboptimal pain care (i.e., direct pathway) and the stress caused by perceived racialized discrimination within and outside pain care (i.e., indirect pathway) (21).

Racism has been conceptualized as a significant threat and stressor for racialized individuals that may detrimentally impact their health (22). In most cases, when we perceive threat, it triggers our stress response system (22). When this system is acutely activated, it may impair our ability to modulate pain and impact our pain threshold and severity (23, 24). However, prolonged activation of the stress response system–often observed among individuals facing racialized discrimination–can dysregulate various bodily responses (e.g., heart rate) and result in epigenetic changes (e.g., changes in gene expression), which may negatively affect overall health (25). Given this, racism–including perceived racialized discrimination–can be viewed as a critical explanatory (i.e., mediating) factor for negative health outcomes, including pain, among racialized individuals (26, 27). Corroborating this notion, Goodin and colleagues (28) found that Black individuals exhibit lower heat pain tolerance compared to non-Hispanic White individuals, and that these lower tolerance levels were predicted by greater perceived racialized discrimination. Similarly, Losin and colleagues (29) demonstrated that heightened pain intensity in Black individuals relative to non-Hispanic White individuals tends to be mediated by perceived racialized discrimination.

While empirical evidence has increasingly pointed at racialized inequities in pain and pain care, critical gaps remain in terms of the populations that have been studied and the contexts in which these inequities have been examined. Indeed, most pain research on racialized inequities has focused on adults; the pain experiences of youth, including their perceptions of racialized discrimination and its potential impact, have garnered considerably less attention [see (7)]. This is unfortunate given that poorly managed pain in childhood contributes to pain problems later in life, as shown by a longitudinal study that followed children aged 8, 11, and 14 into young adulthood (ages 21, 24, or 27), which found that pain experiences in childhood predicted persistent pain in adulthood [see (30)].

Furthermore, most research on racialized inequities in pain and pain care has centered on individuals living in North America [see e.g., (6, 7, 9)], with limited systematic investigation using samples from other parts of the world, including Europe (31). In the Belgian and broader European context, existing health disparities research has largely centered on so-called ‘migrant’ populations, and has tended to frame findings in terms of migration background rather than racialization. While such work provides valuable insights, it risks reproducing racial colorblind narratives that obscure how racism contributes to health inequities. Yet, much like in the United States (U.S.), European societies–including Belgium–are shaped by colonial legacies that continue to affect the lives of racialized individuals through systemic racism.

Taken together, the narrow focus on adults and the overrepresentation of North American samples have hampered our understanding of how racialized inequities in pain and pain care may manifest across different ages and geographical contexts. This is problematic, as it constrains the development of theoretical frameworks that account for the health experiences of racialized individuals across the life course and in diverse cultural settings. In contrast, a better understanding of how racialized inequities in pain and pain care manifest among youth and in European contexts may inform the development of interventions that promote health equity in culturally sensitive and age-appropriate ways. Moreover, addressing racialized inequities in pain and pain care during childhood and adolescence may help prevent long-term health consequences associated with untreated pain [e.g., increased risk of chronic pain and other health problems; (32)], ultimately contributing to a healthier and more equitable society.

To address the above-described knowledge gaps, the current study investigated whether differences exist in diverse pain outcomes (i.e., pain intensity, pain frequency, and pain tolerance) and in perceptions of racialized discrimination in pain care between Black/Brown and White youth living in Belgium. Additionally, we explored whether perceived racialized discrimination in pain care is an explanatory (i.e., mediating) factor in the relationship between participants’ racialized identity and pain outcomes. Drawing on previous research and the RESTORATIVE model (20), which conceptualizes pain inequities as emerging from racism-related trauma across the lifespan, we hypothesized that Black/Brown youth would report greater pain intensity and pain frequency, and exhibit lower pain tolerance compared to White youth (6, 7). Given that Black/Brown individuals are more likely than White individuals to experience racialized discrimination in pain care [(17, 33)], we also predicted that Black/Brown youth would report more perceived racialized discrimination in pain care than their White counterparts.

## 2 Method

### 2.1 Ethical approval

This study was approved by the Ethics Committee of the Faculty of Psychology and Educational Sciences of Ghent University, Belgium.

### 2.2 Data storage

All data and analytic scripts are available on the Open Science Framework website (see https://osf.io/gcu4v/?view_only=2c7f0e10153a425598a6836c12fab34c).

### 2.3 Participants

The current study is part of a larger project that aimed to 1) develop and validate a racially diverse database of posed facial expressions demonstrated by Black/Brown and White children and adolescents; and 2) examine potential disparities in pain experiences and perceived racialized discrimination in pain care among these youth, as well as the interrelationships between these variables. This study only reports findings related to the second objective. A sample of 84 children and adolescents was recruited via social media platforms (i.e., Facebook and Instagram), word-of-mouth advertising, and snowball sampling. This strategy was intentionally implemented to facilitate the recruitment of Black/Brown youth, who are often underrepresented in research. Previous studies have shown that this approach is effective for engaging marginalized populations (34, 35).

Participants were required to meet the following self-reported criteria: being Dutch speaking, self-identifying as White or Black/Brown^1^, being between ages 8 and 17, and having no diagnosed chronic illness, developmental disorder or chronic pain. Specifically, participants were required to be Dutch-speaking to ensure linguistic consistency in the administration and interpretation of the study materials. We included youth who self-identified as either White or Black/Brown in order to examine disparities in pain experiences and perceptions of racialized discrimination in pain care across these groups. The age range of 8 to 17 years was selected because we wanted youth who were able to read and interpret the items in the questionnaires. Furthermore, participants with a diagnosed chronic illness, developmental disorder, or chronic pain were excluded to reduce clinical confounding.

Eight participants were excluded for the following reasons: missing written informed consent (*N* = 6), missing demographics (*N* = 1), and not self-identifying as White or Black/Brown (*N* = 1). Of the remaining participants (*N* = 76, 90.47%), the majority (68.42%) self-identified as White. On average, the sample was 15.17 years old (*SD* = 2.48; *N* < 13 years = 15.79%, *N* > 13 years = 84.21%). The majority of participants self-identified as girls (75%; *N* = 52), while 22.37% (*N* = 17) identified as boys and 2.63% (*N* = 2) as non-binary. Most participants were born (92.11%) and brought up (90.79%) in Belgium. Each participant received €20 for participating in this project.

### 2.4 Materials

#### 2.4.1 Apparatus

The cold pressor apparatus was used to induce pain. It consisted of a commercially manufactured electronic cooler (i.e., cold water tank; W: 35 cm, L: 60 cm, H: 45 cm) that contained cold water that was constantly circulated by a pump to ensure that the water temperature remained at 8°C (± 1°C). To provide comfortable access for each child, the apparatus was placed on a height-adjustable trolley. Next to the cold pressor apparatus was a standardization water tank containing water at room temperature (21°C). Prior research indicates that the cold pressor apparatus is well suited to induce pain that mimics genuine acute pain in youth (36–39).

All self-report assessments were administered via Qualtrics, a web-based platform for creating and analyzing surveys. All questionnaires were completed on a Dell Latitude E5530 laptop.

#### 2.4.2 Self-report measurements

##### 2.4.2.1 Pain intensity and frequency

Participants reported the *pain intensity* they had experienced during the past two weeks on a scale ranging from 0 (no pain) to 100 (worst possible pain), and during the past six months on a scale ranging from 0 (no pain) to 10 (worst possible pain). The *frequency of participants’ pain* over the past six months was assessed by asking them to report the total number of days they experienced pain during this period.

##### 2.4.2.2 Perceived racialized discrimination in pain care

A Dutch version of the Discrimination in Medical Settings scale [DMS; see (40)] was used to assess perceived racialized discrimination in health care. This questionnaire was translated using the forward-backward method (41). All items of the original questionnaire were adapted so that each question assessed participants’ experiences with providers in general (rather than just with doctors and nurses) in the context of pain care (rather than medical care in general). All questions were prefaced with the statement: “When getting pain care, how often has each experience happened to you because of your race or color?” Items (e.g., “You were treated with less courtesy than others”) were answered on a 5-point Likert scale ranging from 1 (never) to 5 (always) and showed excellent internal consistency (Cronbach’s α = .93). Mean scores (ranging from 1 to 5) were calculated, with higher scores indicating more perceived racialized discrimination in pain care.

#### 2.4.3 Pain tolerance

Before measuring participants’ pain tolerance, we standardized their skin temperature by asking participants to place their right hand (up to the wrist) in the tank with water at room temperature for two minutes. Following this standardization phase, participants completed the cold pressor task (CPT). During the CPT, participants were instructed to immerse their right hand (up to the wrist) into the cold water tank until they could no longer endure the pain (i.e., until pain tolerance). Pain tolerance measurement began once participants placed their right hand into the cold water tank. Unbeknownst to participants, a time restriction was in place, so that if participants did not withdraw their hand from the cold water tank after 4 minutes, the researcher terminated the task. During both the standardization phase and the CPT, participants were not informed about the water temperature [see (36, 37)] for similar procedures].

### 2.5 Procedure

Upon arrival, participants and their parents/guardians (if present) were welcomed and informed about the study objectives and procedure. If a participant aged 16 or older provided written consent, or a participant under 16 provided written assent along with parental consent, the experimenter (I.V.A., A.H., E.C., D.D., E.K., or M.D.)^2^ asked the participant to enter the research room without their parents/guardians. There, the participant first completed the questionnaires, after which the CPT was administered in another research room. Once all study phases were completed, participants were thanked and debriefed.

### 2.6 Statistical plan

All continuous variables (dependent and independent) were standardized before analyses, and categorical variables were dummy coded as follows: racialized identity (Black/Brown coded 0, White coded 1); gender (non-binary and boy coded as 0, girl coded as 1)^3^. Due to standardization and dummy coding, the estimated parameters can be interpreted as standardized effect sizes. To assess the effect of racialized identity on pain, first a Multivariate Analysis of Covariance (MANCOVA) was performed with the four pain measures as dependent variables (i.e., pain intensity during the past two weeks, pain intensity during the past six months, pain frequency during the past six months, and pain tolerance). Thereafter, four separate Univariate Analyses of Covariance (ANCOVA) were conducted to examine the relationship between participants’ racialized identity (independent variable) and the pain measures (dependent variables). In both the MANCOVA and ANCOVA, gender and age were included as control variables, as prior research has shown that pain experiences may vary as a function of a child’s gender and age (42, 43). Furthermore, to assess the effect of racialized identity on perceived racialized discrimination in pain care, an ANCOVA was used, with gender and age as control variables.

**Figure 1:**
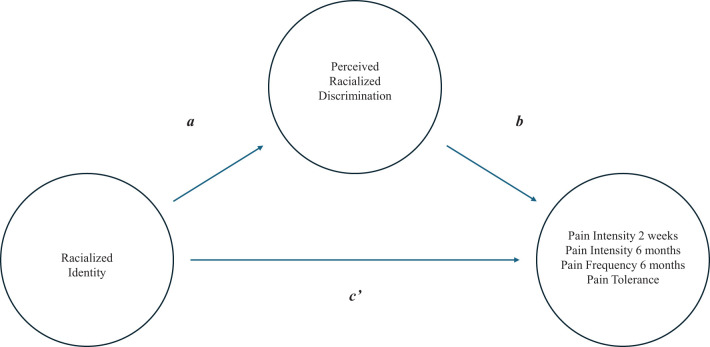
Graphic representation of the mediation model. The total effect (weight *c*) consists of a direct effect (weight *c’*) and the indirect effect (*ab* weight).

To test for mediation, we first made a distinction between the various effects and their corresponding weights (see **Figure 1**). The total effect of racialized identity on a particular pain outcome (weight *c*) comprised: (1) the direct effect of racialized identity on the pain outcome (weight *c’*) and (2) the indirect effect of racialized identity on the pain outcome through a proposed mediator, i.e., perceived racialized discrimination in pain care (weight *ab*). The effect of racialized identity on perceived racialized discrimination in pain care was represented by weight *a*, whereas weight *b* was the effect of perceived racialized discrimination in pain care on the pain outcome, partialing out the effect of racialized identity. To assess the indirect effect, we used a bootstrapping method (i.e., a non-parametric resampling procedure with 5000 bootstrap resamples) (44, 45). Point-estimates and 95% bias-corrected bootstrapped confidence intervals were estimated, with significant mediation demonstrated by bias-corrected bootstrapped confidence intervals that excluded zero.

Analyses were done in R. The *statistics* and *car* packages were used for the MANCOVA and ANCOVAs, and the *Lavaan* package for the mediation analyses. Standardized effect sizes are reported, as well as partial eta squared for the ANCOVA models. Significance was set at *p* <.05. To maintain the familywise error rate for testing differences in pain outcomes, a Bonferroni correction was used, and significance was set at *p* <.05/4 = .0125.

## 3 Results

### 3.1 Descriptives

Overall, participants reported a mean pain intensity of 36.46 (*SD* = 28.01) over the past two weeks (on a scale from 0 [no pain] to 100 [worst possible pain]). Over the past six months^4^, participants experienced pain on an average of 24.43 days (*SD* = 30.04), with a mean intensity of 3.91 (*SD* = 2.16) (on a scale from 0 [no pain] to 10 [worst possible pain]). In the overall sample, the mean pain tolerance was 147.89 seconds (*SD* = 94.51), comparable to levels observed in other samples of children without chronic pain (46). Participants’ mean perceived racialized discrimination in pain care was 1.27 (*SD* = .57; scale range: 1 [never] to 5 [always]). Mean scores and standard deviations for each measure across racialized groups, along with Pearson intercorrelations among the measures, are reported in **Table 1**. To account for multiple comparisons, the *p*-values reported in **Table 1** were Holm-adjusted.

**Table 1:** Means (M), Standard Deviations (SD), and Pearson intercorrelations.

	M_Black/Brown_ (SD_Black/Brown_)	M_White_ (SD_White_)	Age	Pain intensity past 2 weeks	Pain intensity past 6 months	Pain frequency past 6 months	Pain tolerance
Age	14.29 (2.69)	2.30 (2.69)	—				
Pain intensity past 2 weeks	42.71 (27.88)	33.58 (27.87)	.04	—			
Pain intensity past 6 months	4.83 (2.10)	3.48 (2.06)	.13	.58^*^	—		
Number of pain days past 6 months	13.63 (14.65)	29.51 (33.98)	.29	.48^*^	.53^*^	—	
Pain tolerance	92.14 (82.63)	173.62 (88.99)	.30	.00	.08	.25	—
Perceived racialized discrimination	1.77 (.78)	1.04 (.16)	-.17	.30	.06	-.06	-.20

*p <.001. Note that the reported p-values are Holm-adjusted to control for multiple comparisons.

### 3.2 Racialized identity and pain outcomes

Results of the MANCOVA revealed a significant effect of racialized identity on the four pain measures combined (Pillai’s test; *F*(4,68) = 4.77, *p = .*002). No significant effects of youth gender (Pillai’s test; *F*(4,68) = 2.47, *p* = .052) or age (Pillai’s test; *F*(4,68) = 2.36, *p* = .062) were observed.

### 3.3 Racialized identity and pain intensity during the past two weeks

The ANCOVA with pain intensity during the past two weeks showed that, although the direction of the effect was in line with expectations, the effect of youth racialized identity was not statistically significant (*t*(72) = -1.92, *p* = .059, $\eta_{partial}^{2}$ = .026). Furthermore, the standardized coefficient was -.46 (SE = .24), indicating that the average reported pain intensity during the past two weeks was about .46 standard deviations lower for White youth compared to Black/Brown youth. No significant effects of youth gender (*t*(72) = 2.24, *p* = .028, $\eta_{partial}^{2}$ = .077) or age (*t*(72) = -1.75, *p* = .084, $\eta_{partial}^{2}$ = .041) were observed.

### 3.4 Racialized identity and pain intensity during the past six months

The ANCOVA with pain intensity over the past six months did not reveal a significant effect of youth racialized identity (*t*(72) = -1.07, *p* = .288, $\eta_{partial}^{2}$ = .003). However, there was evidence for an effect of youth gender (*t*(72) = 3.06, *p* = .003, $\eta_{partial}^{2}$ = .131). The standardized coefficient for this effect was .76 (SE = .25), indicating that girls reported pain intensity levels over the past six months that were approximately .76 standard deviations higher than those of boys and non-binary individuals. No significant effect of youth age was observed (*t*(72) = .185, *p* = .069, $\eta_{partial}^{2}$ = .045).

### 3.5 Racialized identity and pain frequency during the past six months

The ANCOVA with pain frequency (i.e., number of pain days experienced) during the past six months did not reveal significant effects for participants’ racialized identity (*t*(71) = 1.72, *p* = .090, $\eta_{partial}^{2}$ = .065), gender (*t*(71) = 1.33, *p* = .188, $\eta_{partial}^{2}$ = .032) or age (*t*(71) = 1.37, *p* = .174, $\eta_{partial}^{2}$ = .026).

### 3.6 Racialized identity and pain tolerance

In line with expectations, the ANCOVA with pain tolerance provided evidence for an effect of youth racialized identity (*t*(72) = 3.27, *p* = .002, $\eta_{partial}^{2}$ = .175), with a standardized coefficient of .74 (SE = .23), indicating that Black/Brown youth demonstrated lower pain tolerance that was approximately .74 standard deviations lower than those of White youth. There were no significant effects of youth age (*t*(72) = 2.40, *p* = .019, $\eta_{partial}^{2}$ = .074), or youth gender (*t*(72) =.-1.06, *p* = .292, $\eta_{partial}^{2}$ = .009).

### 3.7 Racialized identity and perceived racialized discrimination in pain care

The ANCOVA with perceived racialized discrimination in pain care showed significantly higher scores among Black/Brown vs. White youth (*t*(72) = -6.86, *p* <.001, $\eta_{partial}^{2}$ = .375). The standardized coefficient for this effect was -1.38 (SE = .20), indicating that White youth reported perceptions of racialized discrimination that were 1.38 standard deviations lower than those reported by Black/Brown youth. There was also a significant effect of youth age (*t*(72) = 2.06, *p* = .043, $\eta_{partial}^{2}$ = .056) with a standardized coefficient of .20, implying that perceived racialized discrimination in pain care increased with age. No evidence for an effect of youth gender was observed (*t*(72) = -.25, *p* = .807, $\eta_{partial}^{2}$ <.001).

### 3.8 Perceived racialized discrimination in pain care, racialized identity, and pain outcomes

We further explored the mediating role of perceived racialized discrimination in the relationship between racialized identity and pain outcomes. The mediation model for the four respective outcomes is depicted in **Figure 1**. Results of the mediation analyses were mixed. A significant indirect effect of racialized identity on pain intensity over the past two weeks via perceived racialized discrimination in pain care was found (z = -2.56, p = .010). This finding suggests that differences between White and Black/Brown youth in pain experienced over the past two weeks may be partially explained by differences in their perceived racialized discrimination in pain care. The standardized coefficient for this indirect effect (ab) was -.374 (SE = .146; 95% CI [-.714, -.128]). However, no indirect effects were found for the other three pain outcomes, as all bootstrap confidence intervals included zero.

## 4 Discussion

Empirical evidence has highlighted racialized inequities in pain and pain care, yet the experiences of youth, particularly in Europe, have been largely overlooked in this research. This study aimed to address this gap by examining differences in pain outcomes (i.e., pain intensity, frequency, and tolerance) as well as perceived racialized discrimination in pain care between Black/Brown and White youth in Belgium. Additionally, we explored whether perceived racialized discrimination mediated the relationship between racialized identity and pain outcomes. Findings indicated that, compared to White youth, Black/Brown youth demonstrated lower pain tolerance and reported greater perceived racialized discrimination in pain care. No significant group differences were observed in participants’ self-reported pain intensity during the past two weeks and six months, nor in pain frequency during the past six months. Furthermore, perceived racialized discrimination in pain care mediated the relationship between racialized identity and pain intensity over the past two weeks, but did not mediate the relationships between racialized identity and either pain intensity or pain frequency over the past six months, or pain tolerance.

As hypothesized, and consistent with prior research [e.g., (7, 8)], Black/Brown youth exhibited lower pain tolerance compared to White youth. Building on previous research, we propose that this lower pain tolerance among Black/Brown youth may stem from a combination of physiological, psychological, and socio-cultural factors. Research has identified racialized differences in how cortisol, β-endorphin, and allopregnanolone interact with the hypothalamic-pituitary-adrenal (HPA) axis to influence pain experiences, suggesting that some endogenous pain regulation systems operate less efficiently in African Americans compared to White individuals (11). These physiological differences are shaped by chronic exposure to stressors (e.g., systemic racism), which dysregulate stress-response pathways over time, ultimately contributing to negative health outcomes (including pain) in racialized children and adults (25, 47). Additionally, prior evidence indicates racialized differences in pain catastrophizing, with Black individuals generally reporting higher levels than White individuals (10). These heightened levels of catastrophizing have, in turn, been found to mediate racialized disparities in pain tolerance, with greater catastrophizing being associated with lower pain tolerance among Black individuals (48, 49). Furthermore, research shows that pain perception is shaped not only by individual (i.e., psychological and physiological) mechanisms, but also by sociocultural factors. Specifically, existing evidence indicates that cultural beliefs, values, and practices may influence how pain is experienced and managed. For instance, cultural norms that valorize stoicism or encourage expressiveness can shape whether individuals display or suppress pain (50). In this light, the observed differences in pain tolerance during the CPT may, at least partially, reflect differential adherence to culturally shaped norms around pain endurance. That said, it is important to acknowledge that considerable cultural variability exists within racialized groups, even though shared experiences of racialization (e.g., discrimination, stereotyping) may give rise to certain common cultural norms, including those related to pain tolerance.

Taken together, differences in cultural norms, pain catastrophizing, and endogenous pain modulation may have contributed to the lower pain tolerance observed in Black/Brown youth compared to White youth in the present study. However, as we did not directly assess these mechanisms, these explanations remain tentative and should be examined in future research. Future work could also explore the role of other potential contributors, such as social factors (e.g., the presence and characteristics of another person; see infra), to racialized disparities in pain experiences. Research that examines the explanatory role of physiological, psychological, cultural, and social factors may contribute to a more comprehensive understanding of these disparities.

Notably, in contrast to the racialized disparities in pain tolerance, we did not find statistically significant differences between Black/Brown and White youth on self-reported pain intensity during the past two weeks or six months, or on pain frequency during the past six months. The mean values do, however, suggest potentially meaningful differences, particularly on pain intensity during the past two weeks, with Black/Brown youth reporting higher pain intensity compared to White youth. Although not statistically significant, the direction and magnitude (*d* = .33, small-moderate) of this difference is roughly similar to those from population-based studies of U.S. adults (51). Our small sample size and unbalanced groups may have contributed to the lack of statistical significance of this group difference.

Besides these considerations, it is also possible that biases in participants’ self-reported pain may have contributed to this lack of statistical significance. Evidence suggests that self-reported pain can be prone to various biases, including recall bias and response bias. Specifically, research indicates that individuals often struggle to accurately recall past pain experiences (52, 53), finding it particularly difficult to remember the intensity or frequency of pain, but finding it easier to remember its location (54). Self-reported pain has also been found to be susceptible to response biases, such as socially desirable responding, where individuals modify their responses to align with perceived social norms or expectations (55). Moreover, empirical research shows that both racialized and White children hold the belief that Black children feel less pain than their White counterparts (56, 57). This belief, which has historical roots in colonialism and slavery (57), reflects a racialized bias about pain experiences that - if internalized - could lead Black individuals to underreport their pain. Taken together, these biases may have contributed to an underestimation of racialized participants’ actual pain experiences, potentially reducing the likelihood of detecting statistically significant racialized disparities in pain intensity and frequency in our study.

Our finding that racialized differences in pain tolerance were more robust than racialized differences in self-reported pain is consistent with prior research suggesting that such disparities are especially apparent in pain behaviours [see (58)]. Nevertheless, other studies show that racialized disparities extend beyond pain tolerance to self-reported pain intensity and frequency, though results remain mixed. For instance, both experimental and clinical research have documented higher self-reported pain intensity among Black individuals compared to their White counterparts (7, 11). In contrast, population-based research in the U.S. has reported that White individuals experience more frequent or persistent pain than Black individuals (59, 60). Complicating matters further, other studies have found no racialized differences in pain frequency among healthy young adults (61). It is not clear where to situate the current findings in this broader literature, given that much of this research has focused on adults, while studies on racialized disparities in pain among youth remain comparatively limited [see (7)]. This gap, combined with the inconsistencies in the existing literature, further highlights the need for additional research on racialized disparities in pain outcomes and its potential underlying mechanisms among youth.

Consistent with our hypotheses and prior research [e.g., (62)], Black/Brown youth reported significantly greater perceived racialized discrimination in pain care. This finding aligns with existing evidence of racialized inequities in Belgian healthcare (63) and research indicating that, under some circumstances, Black adolescents experience racialized discrimination and stigma when accessing pain care for acute and chronic pain (33, 64–66). Notably, our study is the first to document greater perceived racialized discrimination in pain care among Black/Brown youth compared to White youth in Belgium, demonstrating that these inequities extend beyond the U.S. context. Further research is therefore needed to better understand how racialized pain care inequities are experienced by youth in Belgium, in order to assess the robustness of our findings. This includes work on the mechanisms through which such inequities are produced and maintained. One factor often viewed as an important explanatory mechanism is healthcare providers’ implicit racialized attitudes–biased assumptions about racialized groups (e.g., that Black people feel less pain) that are activated relatively automatically (67). However, while some studies have found associations between these attitudes and clinical decision-making in pain care, others have not (68–70). Given these mixed findings and the limited research on the explanatory mechanism of racialized inequities in Belgian pediatric pain care, further work should explore the conditions under which implicit bias and other factors are associated with and give rise to racialized inequities.

Finally, we explored whether perceptions of racialized discrimination could help explain (i.e., mediate) racialized differences in pain outcomes (i.e., pain intensity over the past two weeks and six months, pain frequency over the past six months, and pain tolerance). This exploration was informed by previous research showing that perceived discrimination predicted pain tolerance for Black but not White participants (28), and mediated higher pain reports among Black participants compared to White participants (29). However, our analyses revealed mixed evidence for the mediating role of perceived racialized discrimination in the relationship between racialized identity and pain outcomes. Specifically, we found that perceived racialized discrimination in pain care mediated the relationship between racialized identity and pain intensity during the past two weeks, which aligns with existing literature suggesting that racism–rather than racialized identity itself–helps explain observed health disparities (21, 25, 71). Nevertheless, these perceptions did not significantly mediate the relationship between racialized identity and the other three pain outcomes.

Several factors may explain why perceived racialized discrimination did not significantly mediate the relationship between racialized identity and the other three pain outcomes. To begin with, the present study may have been underpowered to detect mediation relationships. A *post-hoc* sensitivity analysis indicated that a sample size of at least 190 (more than double our sample size of 76) would have been required to detect the observed effect with 80% power. With this in mind, we caution against overinterpreting our mediation findings. Future research with larger samples is needed to more adequately examine the mediating role of perceived racialized discrimination in the relationship between racialized identity and pain outcomes.

Besides sample size limitations, we may have failed to detect additional mediation effects because our sample consisted of individuals without clinical pain, who typically seek healthcare less frequently than those with clinical pain. As a result, our study participants may have had fewer opportunities to encounter discrimination in healthcare settings, thereby weakening the effect of perceived racialized discrimination in pain care on their pain outcomes. This reduced exposure to discrimination in healthcare contexts among non-clinical samples may partly explain why prior research has found strong links between healthcare discrimination and pain outcomes among minoritized clinical populations (72, 73), whereas our study found only limited evidence for this relationship.

Lastly, it is important to acknowledge that racialized discrimination can occur across multiple domains, including law enforcement, hospitality services, and education, in addition to healthcare (62, 63). Repeated exposure to these stressors can, over time, dysregulate the body’s stress-response systems, which in turn may impair pain modulation and exacerbate pain outcomes (22, 64). Because our study focused solely on healthcare-related discrimination–just one part of a broader landscape of systemic racism–we may have captured only a small portion of the overall impact of racialized discrimination. A more comprehensive measure, incorporating racialized discrimination across multiple domains, might have revealed more mediation effects.

The present study has several notable strengths. We recruited a pediatric and European sample, both of which represent understudied populations in the pain disparities literature. Additionally, we incorporated both self-report and behavioral measures of pain outcomes, providing a more comprehensive understanding of participants’ pain experiences. However, several limitations warrant consideration. First, as stated earlier, our sample was relatively small, which hampers the generalizability of our findings. In addition, as participants were recruited via social media, word-of-mouth advertising, and snowball sampling, some degree of selection bias may have occurred. Future research with larger samples is warranted to assess the robustness of our findings. Second, the cross-sectional design hampers our ability to make causal inferences about the relationship between racialized discrimination and pain outcomes. Future longitudinal studies are needed to capture these dynamics and elucidate potential causal pathways. Third, as previously mentioned, the use of a non-clinical sample may have limited the variability in our pain outcomes, further impacting our ability to detect significant relationships between perceived racialized discrimination and pain outcomes. Fourth, the exclusive focus on White or Black/Brown youth aged 8–17 restricts the generalizability of our findings to other racialized identities and age groups. Future studies should include a broader range of racialized populations and age groups to explore potential similarities and differences in pain experiences and the quality of pain care they receive. Fifth, while we controlled for age and gender in our analyses, we did not account for how intersecting systems of oppression and power (e.g., racism and ableism), shaped by individuals’ social identities, may influence pain experiences. We recommend that future research examines these intersections more explicitly to advance our understanding of pain inequities. Sixth, this study did not incorporate objective markers of racialized discrimination in pain care, such as disparities in pain treatments documented in medical records. Including both subjective experiences and objective data in future research would allow for a more comprehensive understanding of these inequities. Seventh, we did not examine how the presence of the experimenter or their characteristics (e.g., gender and racialized identity) influenced participants’ pain tolerance. Prior research suggests that the presence of another person during a cold pressor task can increase pain thresholds compared to completing the test alone, and that this effect may vary by the observer’s characteristics (e.g., gender) (23). We recommend that future research investigates the influence of the presence and characteristics of the experimenter on pain tolerance outcomes. Eighth, as previously mentioned, the use of self-report measures may have introduced recall and reporting bias. Future research could minimize these biases by employing diary methods to assess pain experiences in real time and include multiple informants (e.g., youth and their parents or guardians) to specifically address potential reporting bias. Finally, while the Discrimination in Medical Settings scale has shown to be valid (40), it should be noted that we modified this scale to refer specifically to pain care. Future research is therefore needed to assess its validity.

The present study expands our understanding of racialized disparities in pain outcomes and perceived racialized discrimination in pain care, particularly among youth in Europe–a population that has been largely overlooked in prior research. The findings highlight the complex interplay between racialized identity, perceived racialized discrimination in pain care, and pain outcomes. By shedding light on these relationships, this study offers valuable insights that underscore the importance of more inclusive and equitable pain care.

## Data Availability

The datasets presented in this study can be found in online repositories. The names of the repository/repositories and accession number(s) can be found below: https://osf.io/gcu4v/?view_only=2c7f0e10153a425598a6836c12fab34c.

## Footnotes

1 Participants’ self-identified racialized identity was assessed by asking them to select their skin color from the following options: white, black, brown, or another skin color.

2 The initials represent the experimenters: two White men, three White women, and one Black woman. See the acknowledgement section for their full names.

3 Given the small number of non-binary participants, only one dummy variable was used for gender. To assess the robustness of the results, all analyses were repeated with an alternative dummy for gender (non-binary and girl coded as 0, boy coded as 1). Similar results were obtained and as such, the study’s conclusions were not affected.

4 Note that there was one missing observation for this variable.
